# Remarkable response to anti-PD1 immunotherapy in refractory metastatic high-grade myxofibrosarcoma patient: A case report

**DOI:** 10.1097/MD.0000000000025262

**Published:** 2021-03-26

**Authors:** Yi Luo, Li Min, Yong Zhou, Fan Tang, Minxun Lu, Hongmei Xie, Yitian Wang, Hong Duan, Wenli Zhang, Chongqi Tu

**Affiliations:** a Department of Orthopedics, West China Hospital, Sichuan University, Chengdu, Sichuan, P. R. China; b Yinfeng Gene Technology Co Ltd, CORA.

**Keywords:** anti- programmed cell death protein 1, Camrelizumab, immunotherapy, myxofibrosarcoma

## Abstract

**Introduction:**

Myxofibrosarcoma (MFS) is a locally aggressive tumor and has the potential to be fatal because of distant metastasis. Immunotherapy targeting either programmed cell death protein 1 (PD-1) or programmed death ligand 1 (PD-L1) has recently shown a curative effect on multiple cancers including melanoma, non-small cell lung cancer, and renal cell carcinoma. Although the immunotherapy has been applied in sarcoma, there is little information about the efficiency to treat metastatic MFS.

**Patient concerns:**

A 42-year-old male presented to the clinic with a mass in the left thigh. Mass resection and ligament replacement surgery were performed.

**Diagnoses:**

The patient was diagnosed as high-grade MFS (federation nationale des centres de lutte contre le cancer, Grade 3) with pulmonary metastasis.

**Interventions:**

In the past few years, he was treated with surgery, chemoradiotherapy, and Anlotinib (an angiogenesis inhibitor), but the metastatic lesion continued to progress. About 40% to 50% of tumor cells in his pulmonary tissues were showed positive PD-L1 expression and his tumor mutational burden was 215Muts. Thus, he received Camrelizumab (PD-1 inhibitor).

**Outcomes:**

Six months after the initiating immunotherapy of Camrelizumab, the size of pulmonary lesions showed marked shrinkage, indicating a partial response. After a follow-up of 18 months, the patient remained in good condition without progressive disease.

**Conclusion:**

This case described here demonstrated that immunotherapy of PD-1 inhibitor is a promising treatment option for refractory MFS with PD-L1 positive or tumor mutational burden -high, which could contribute to effective tumor response.

## 1 Introduction

Myxofibrosarcoma (MFS) is a unique subtype of soft tissue sarcoma typically arising as a painless slow-growing mass in the extremities, representing around 5% of soft tissue sarcoma. According to the 3-tiered Federation Nationale des Centres de Lutte Contre le Cancer (FNCLCC) system, MFS is divided into 3 grades. High-grade myxofibrosarcoma characterizing as high mitotic rate and necrosis, is more likely to recur and metastasize.^[1]^ The recurrence rate of MFS is from 16% to 57%, which is higher than those of other soft tissue sarcomas.^[2]^ And 15% to 38% of MFS with local relapse could develop into distant metastasis.^[3]^

Margin-negative surgical resection of the mass is the most common treatment for myxofibrosarcoma patients. However, a significant proportion of patients can’t benefit from surgery due to the high risk of local recurrence and distant metastasize. They continue to die from the metastatic disease despite operation combined with radiotherapy and/or chemotherapy.

Immunotherapy is a novel approach for tumor treatment. In tumors, cells highly express programmed death ligand-1 (PD-L1), and over- expressed PD-L1 mediates evasion of tumor cells from the immune system through binding to programmed cell death 1 (PD-1) protein expressed by activated immune cells.^[4]^ PD-1 blockades can fight against cancer cells through suppressing PD-1 binding to PD-L1 to reboot the immune system. Up to now, a series of monoclonal antibodies have been approved for cancer immunotherapy, such as pembrolizumab, nivolumab, and Camrelizumab. A lot of evidence from various clinical trials and reported cases confirmed blockade of PD-L1 can cause an efficient response in patients with advanced melanoma, lung cancer, renal cell carcinoma, and gastric cancer. A multicenter, open-label phase 2 trial included the treatment of soft tissue sarcoma with nivolumab offered no evidence of clinical activity in soft tissue sarcoma containing 2 patients with MFS.^[5]^ Nonetheless, another phase 2 trial informed 7 (18%) of 40 patients with soft-tissue sarcoma showed an objective response to pembrolizumab.^[6]^ As a subset of soft tissue sarcoma, a few cases of MFS treated with PD-1 blockades are reported. Hence, a unique patient of metastatic high-grade myxofibrosarcoma with marked response to the administration of PD-1 blockade was presented. To our limited knowledge, this is the first report on the treatment of Camrelizumab in patients with MFS.

## 2 Case presentation

A 42-year-old male presented to the clinic with a mass in the left thigh in August 2017. Mass resection and ligament replacement surgery were performed on November 7, 2017. The postoperative pathological examination result showed a high-grade myxofibrosarcoma (FNCLCC, Grade 3). However, retropleural nodules were found in his right upper lode a few months later. Hence, extended-field radiotherapy with a radiation dose of 50Gy/25F/35d was carried out. Then the patient received 3 cycles of chemotherapy with cisplatin, ifosfamide and epirubicin. Stable disease was obtained during this period.

**Figure 1:**
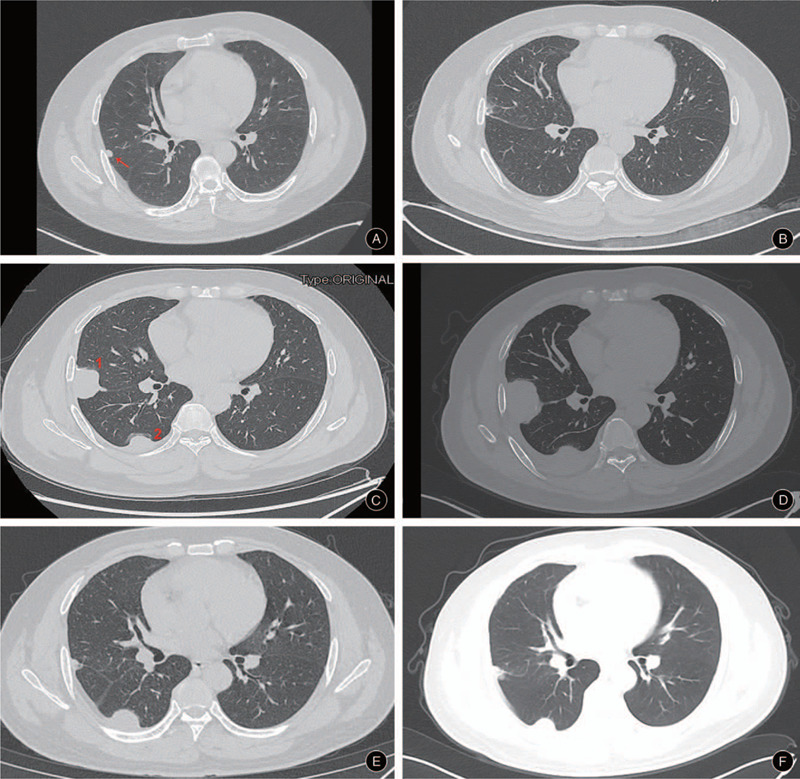
(A) The pulmonary lesion in the right upper lode scanned through CT on July 1, 2018; (B) The right upper lode after the thoracoscopic pulmonary nodule resection (August 15, 2018); (C) The pulmonary lesion relapsed 4 months after the pulmonary nodule resection (November 23,2018) and lesion 1 and 2 were 38.8 mm × 24.8 mm and 14.2 mm × 9.2 mm in size, respectively; (D) The pulmonary lesion enlarged 1 month after the initial administration of Anlotinib (December 22, 2018) and lesion 1 and 2 were 49.3 mm × 34.2 mm and 37.0 mm × 20.2 mm in size, respectively; (E) The pulmonary lesion decreased 5 month after the initial administration of Camrelizumab (June 2, 2019) and lesion 1 and 2 were 8.4 mm × 8.0 mm and 16.6 mm × 13.4 mm in size, respectively; (F) The pulmonary lesion scanned 12 month after the initial administration of Camrelizumab (January 2, 2020) and lesion 1 and 2 were 12.4 mm × 9.0 mm and 16.5 mm × 13.2 mm in size, respectively.

Two months after chemotherapy, a follow-up chest computed tomography (CT) scan (July 1, 2018) depicted enlarged nodules, indicating disease progression (Fig. 1A). Therefore, the patient underwent thoracoscopic pulmonary nodule resection in July 2018 (Fig. 1B). The lung lesion was confirmed as metastatic myxofibrosarcoma according to the consequence of the postoperative pathological examination. However, metastases with a maximum lesion of 38.8 mm × 24.8 mm and a minimum lesion of 14.2 mm × 9.2 mm were screened on November 23, 2018 (Fig. 1C). The administration of the target drug Anlotinib was carried out. However, the tumor showed no response to Anlotinib, and lesion 1 and lesion 2 raised to 49.3 mm × 34.2 mm and 37.0 mm × 20.2 mm in size, respectively, indicating a serious disease progression (Fig. 1D).

**Figure 2:**
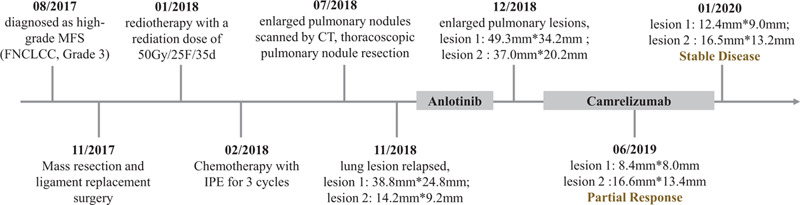
Schematic picture showing the time line of treatment procedure.

For further treatment, detections related to immunotherapy were tested. On immunohistochemistry, about 40% to 50% of tumor cells showed high PD-L1 expression. Meanwhile, whole-exome sequencing (tested by Yinfeng Gene Technology Co., Ltd.) was performed and his microsatellite instability status was stable (MSS) and tumor mutational burden (TMB) was 215Muts. Therefore, PD-1 inhibitor (Camrelizumab, Jiangsu Hengrui Medicine Co. Ltd) was administrated by intravenous drip (started on January 11, 2019), at a dose of 200 mg, every 3 weeks. After 3 courses of treatment, the symptom of cough was remitted completely. Of note, CT displayed reduction in lesion 1 from 49.3 mm × 34.2 mm to 8.4 mm × 8.0 mm and lesion 2 from 37.0 mm × 20.2 mm to 16.6 mm × 13.4 mm (Fig. 1E). At his last follow-up, 12 months after his initiating immunotherapy of Camrelizumab, the size of lesion 1 (12.4 mm × 9.0 mm) and lesion 2 (16.5 mm × 13.2 mm) were stable (Fig. 1F), and the patient was observed in good condition, exhibiting a sustained durable response without any adverse events (Fig. 2).

## 3 Discussion

Myxofibrosarcoma, as a malignant soft-tissue tumor, remains a high rate of local recurrence and distant metastasis. Under the FNCLCC system, myxofibrosarcoma must exhibit both high mitotic rate and necrosis to achieve a designation of high-grade.^[1]^ There are multiple locations of distant metastasis in myxofibrosarcoma, in which lung metastases are the most common metastatic site. A retrospective study reported 16 myxofibrosarcoma patients with distant metastases, and 11 of which developed pulmonary metastases.^[7]^ Our patient introduced here, local recurrence in the left thigh bottom was observed 2 years after the initial surgery. And pulmonary metastases were found several months after the second surgery. Afterward, lung nodules substantially grew in size and number screened by chest CT scan.

Surgical operation followed by radiotherapy and/or chemotherapy is recommended treatment for myxofibrosarcoma. And a positive margin is a predictor of poor prognosis. For patients with positive margin, the 5-year re-recurrence free survival was only 9.8% compared with that of 62.3% in patients with negative margin.^[8]^ Adjuvant radiotherapy is taken as a standard treatment for operable patients with MFS, and palliative chemotherapy is applied in selective patients with metastatic sites.^[9]^ Regrettably, the metastatic lesion in the lung of our patient appeared no response to the combined therapies. From a series analysis of 56 consecutive patients with MFS, the author deemed improved tumor control by the administration of radio- and/or chemotherapy couldn’t be demonstrated.^[2]^ It suggests that not all patients with metastatic MFS can benefit from chemotherapy and/or radiotherapy after mass resection.

Nowadays, PD-1 blockades are widely used in multiple tumor types and the effective response has been obtained. However, a significant portion of patients can’t gain hope from PD-1 inhibitors and showed no response. The choice of suitable patients for PD-1 inhibitors appears especially important. PD-L1 expression level on tumor cells measured by immunohistochemistry was introduced as a predictor. Numerous investigations of PD-L1 expression in different tumors were carried out. Whereas, the expression status of PD-L1 in MFS remained controversial. Kosemehmetoglu K et al reported a negative PD-L1 expression in MFS.^[10]^ On the contrary, Jan Budczies et al demonstrated PD-L1 copy number gains were most prevalent in MFS compared with other subtypes.^[11]^ In our case, 40% to 50% of tumor cells were stained positively by immunohistochemistry and a partial remission was observed. A special patient who had double neoplasms with advanced NSCLC and mediastinal MFS without PD-L1 expression was reported. In the course of processing lung cancer with pembrolizumab, the mediastinal tumor enlarged extremely rapidly from 5 cm to 15 cm in size within 3 months.^[12]^ Therefore, patients with PD-L1 over-expression may potentially benefit from PD-1 inhibitors.

Tumor mutational burden (TMB), representing the number of somatic mutations in a tumor that form neoantigens, is responsible for the immunogenicity of tumors. TMB is an emerging predictor for predicting immunotherapy efficacy and associated with a better response to immunotherapy. Recently, pembrolizumab was approved by the US Food and Drug Administration (FDA) for the indication of a solid tumor with TMB-H (≧10muts/Mb) in adults and children. In this report, TMB was detected as TMB-high with a value of 215Muts by next-generation sequencing (NGS). Nevertheless, the value of TMB in varied tumors is different. TMB of undifferentiated pleomorphic sarcoma (UPS) was detected in a study enrolled in 16 patients with recurrence, in which the average value was 7.5 Muts and the highest 1 was nearly 30 Muts.^[13]^ Another analysis of 100,000 human cancer genomes revealed that the average and maximum value of TMB were 2.5 Muts and 26.7 Muts in bone osteosarcoma (n = 283), as well as 2.2 Muts and 145.2 Muts in soft tissue myxofibrosarcoma (n = 58), respectively.^[14]^

Regarding to MFS treated with PD-1 blockades, rare cases were reported. Haa-Na Song et al^[15]^ presented a patient with conventional chemotherapy-refractory metastatic MFS was administrated with pembrolizumab as palliative immunotherapy and it achieved a partial response. Another case with double neoplasms with advanced NSCLC and a mediastinal myxofibrosarcoma was also treated with pembrolizumab and no recurrence was found before the patient died 8 months after surgery.^[12]^ A phase 2 trials enrolled 1 patient with chemotherapy- refractory MFS receiving nivolumab obtained confirmed response.^[16]^ To the best of knowledge, this case represents the first evidence of Camrelizumab exhibiting efficacy against refractory metastatic MFS without any toxicity.

## 4 Conclusion

In conclusion, a unique case of myxofibrosarcoma (FNCLCC, Grade 3) with severe lung metastasis was described here. The patient was administrated with anti-PD1 immunotherapy and a partial response was obtained. Therefore, for severely metastatic myxofibrosarcoma with no response to conventional therapeutic options, immunotherapy is a promising selection.

## Acknowledgments

We would like to thanks for the help of Yinfeng Gene Technology Co., Ltd. in organizing the data and revising this article.

## Author contributions

**Conceptualization**: Yi Luo.

**Data curation**: Li Min, Yong Zhou, Fan Tang.

**Investigation**: Minxun Lu, Yitian Wang.

**Supervision**: Hong Duan.

**Validation**: Wenli Zhang, Yong Zhou, Chongqi Tu.

**Writing – original draft**: Chongqi Tu, Hongmei Xie.

**Writing – review & editing:** Hongmei Xie.
